# A synthetic metabolic pathway for the de *novo* biosynthesis of medium chain length γ- and δ-lactones

**DOI:** 10.1186/s13036-025-00575-z

**Published:** 2025-11-25

**Authors:** Jonathan Muller, Angel Xia, Patrik Jones

**Affiliations:** Department of Life Sciences, Imperial College London, London, UK

**Keywords:** γ-decalactone, Lactones, Thioesterase, BM3, Metabolic engineering, Synthetic biology

## Abstract

**Background:**

Medium chain length γ- and δ-lactones, in particular γ-decalactone, are potent aroma compounds used by the flavour and fragrance industry. The native pathways for their biosynthesis are unknown so to facilitate future biomanufacturing, we designed and implemented a novel metabolic pathway, using engineered enzymes, for the *de novo* biosynthesis of lactones, leading to the production of γ-decalactone, δ-decalactone, γ-dodecalactone and δ-dodecalactone in *Escherichia coli*.

**Results:**

Wild-type enzymes with the appropriate substrate specificities for the pathway were not available, therefore enzyme engineering was required. Firstly, the *Cuphea viscosissima* FatB1 thioesterase was modified to be C10 specific, resulting in novel mutants with a 77.1% C10 specificity in *E. coli*. Engineered thioesterases were also found to display increased C10 specificities when expressed in *Synechocystis* Sp. PCC 6803. The cytochrome P450 BM3 was then used to hydroxylate decanoic acid to 4- or 5-hydroxydecanoic acid which spontaneously condensed to γ- and δ-decalactone, respectively. Feeding methyl decanoate to *E. coli* cells expressing BM3 mutants led to improved production of both γ- and δ-decalactone. Expressing the complete pathway using the engineered enzymes enabled the accumulation of γ- and δ-decalactone with maximum titres of 3.53 mg/L and 0.51 mg/L, respectively. The pathway was also modified to biosynthesise dodecalactones by using a C12-specific thioesterase and different BM3 mutants leading to γ- and δ-dodecalactone titres of 1.21 mg/L and 3.29 mg/L, respectively.

**Conclusions:**

The synthetic pathways were shown to be functional and amenable to tailoring both the chain length and ring-structure of the resultant lactones.

**Supplementary information:**

The online version contains supplementary material available at 10.1186/s13036-025-00575-z.

## Background

Medium chain length γ- and δ-lactones are potent aroma compounds found in more than 120 foodstuffs, such as fruit, and are also used in the flavour and fragrance industry [1–4]. The native biosynthetic pathways for γ- and δ-lactones are not well understood, and while it is known that 4- and 5-hydroxyfatty acids are precursors to γ- and δ-lactones biosynthesis, how the precursors themselves are biosynthesised is unknown.

The most widely produced and commercially important lactone is γ-decalactone [5]. In isolation, γ-decalactone has an odour described as peach-coconut, and is an important component of peach, apricot and strawberry aroma [6–9]. Global demand for γ-decalactone totals several hundred tons annually [10]. It has a high market value, with the first microbially synthesised γ-decalactone worth €10,000 /kg. After optimisation of the manufacturing process, the price has now dropped to $300 /kg [7, 11]. The closely related δ-decalactone has a similar aroma to γ-decalactone, and is described as having an intensely fruity, peach-coconut odour [9, 12].

Previous reports of the biosynthesis of γ-decalactone used *Yarrowia lipolytica* to biotransform ricinoleic acid (12-hydroxy-9-octadecenoic acid) or methyl ricinolate to γ-decalactone [13–16]. The fed substrate enters the β-oxidation pathway [8], and after multiple rounds of the β-oxidation cycle generates 4-hydroxydecanoyl-CoA. It is not clear how γ-decalactone is thereafter produced from 4-hydroxydecanoyl-CoA.

This concept of feeding a long-chain fatty acid substrate into the β-oxidation cycle has since been expanded to other fatty acid substrates and host species, with a range of medium chain length γ- and δ-lactones being produced [2, 4, 17–22]. *De novo* biosynthesis of lactones has also been reported by engineering the native fatty acid biosynthesis of *Ashbya gossypii* to overproduce linoleic acid, followed by oxidation in the β-oxidation pathway, to produce a range of γ-lactones [15].

Alternatively, Cytochrome P450s (CYP450s) have been used to hydroxylate decanoic acid and dodecanoic acid at the C5 position, with the resulting hydroxyfatty acids condensing under acidic conditions to form δ-decalactone and δ-dodecalactone [23, 24].

**Fig. 1:**
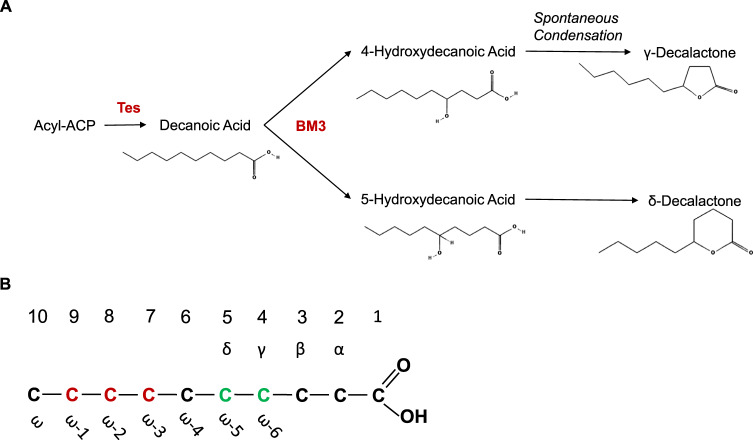
Proposed pathway for biosynthesis of γ- and δ-decalactone **A**) schematic overview of the pathway. Pathway enzymes are highlighted in red. Tes: C10 AcylACP thioesterase, BM3: CYP450 CYP102A1 (*Priestia megaterium*) **B**) regioselectivity of BM3. A schematic diagram of decanoic acid is shown, with the carbons numbered, α to δ carbons and ω to ω-6 carbons labelled. BM3 natively hydroxylates the carbons in red, but in order to produce the hydroxyfatty acid precursors to γ- and δ-decalactone the carbons highlighted in green need to be hydroxylated

In this work, a novel metabolic pathway for the biosynthesis of γ- and δ-decalactone is proposed. The pathway is comprised of a C10 acyl-ACP thioesterase to produce free decanoic acid from endogenous fatty acid biosynthesis. A CYP450 is then used to hydroxylate the decanoic acid at the 4 or 5 position. The 4- or 5-hydroxydecanoic acids thereafter spontaneously condense to respectively form γ- and δ-decalactone, which has previously been reported in the literature [23] (Fig. 1a).

The first step in the proposed biosynthetic pathway is catalysed by an acyl-ACP thioesterase. These enzymes terminate fatty acid biosynthesis by hydrolysing the thioester bond between ACP and the growing acyl chain [25]. Different thioesterases have different specificities for acyl-ACP of various chain lengths. To biosynthesise decalactones, decanoic acid will first need to be produced, and to do so a C10 specific thioesterase will be required.

Microbial acyl-ACP thioesterases tend to be active across a wide range of acyl-ACP chain lengths while plant acyl-ACP thioesterases are generally more specific [26, 27]. Plant thioesterases are classified into two main families, FatA and FatB. FatA acyl-ACP thioesterases are specific for unsaturated C18 acyl-ACP, while FatB acyl-ACP thioesterases are specific for unsaturated acyl-ACP of chain lengths ranging from C8 to C18 [27, 28]. While many FatB thioesterases have been reported [27–43], only seven of them have displayed a prominent medium chain length specificity [25, 27, 33, 44–46]. Of these medium chain length specific acyl-ACP thioesterases, only C4, C8 and C12 specific thioesterases had been reported. The *Cuphea viscosissima* FatB1 (CvFatB1) thioesterase had the highest reported C10 specificity of any wild-type thioesterase in the literature, however it still displayed a preference for C8 over C10 (51.7% vs 25.7%) [27]. This lack of a wild-type C10 thioesterase thus prompted us to engineer a C10 specific acyl-ACP thioesterase in order to maximise chances for decalactone biosynthesis.

It should be noted that after this engineering had been completed, the acyl-ACP thioesterase FatB3 from *Cuphea lanceolata* was reported to be C10 specific (70% specificity) when expressed in *E. coli* [46].

FatB thioesterases have N-terminal chloroplast localisation peptides, that need to be excised to form the active enzyme [28, 30]. While it is known that this excision, hereafter referred to as truncation, is required, the ideal site of truncation for FatB thioesterases is unknown. It has been reported that truncating the same thioesterase at different locations leads to different activity and specificity profiles [47–49], and so optimisation of the location of truncation is useful tool for optimising titres and product specificities when expressing FatB thioesterases in bacteria.

FatB thioesterases have previously been engineered with altered activity and specificity. Through random mutagenesis, a highly active and C8 specific variant of *Cuphea palustris* FatB1 was obtained, which had a unique truncation and two point mutations [48]. *Cuphea hookerina* FatB2 was engineered to be more C10 specific by incorporating a point mutation that modulated the interactions between the thioesterase and ACP. While this increased the proportion of decanoic acid out of total fatty acids produced, the total fatty acid production was reduced [50]. Another approach has been to alter the specificity by engineering the binding pocket, either by introducing residues from another FatB thioesterase with a different chain-length specificity or by mutating residues to enlarge the binding pocket, and this approach has been used several times to generate mutant FatB thioesterase with increased specificity for longer chainlength substrates [41, 51, 52].

The second step in the proposed decalactone biosynthesis pathway is the hydroxylation of decanoic acid at the 4- and 5-positions, to form the appropriate 4- and 5-hydroxydecanoic acid precursors to γ-decalactone and δ-decalactone.

CYP450 BM3 or CYP102A1 (BM3) is a soluble catalytically self-sufficient monooxygenase from *Priestia megaterium* (formerly called *Bacillus megaterium* [53]). BM3 natively hydroxylates C12 to C20 fatty acids at the ω-1, ω-2 and ω-3 positions in a highly regioselective manner [54–56]. BM3 has the highest reported catalytic activity for a P450 monooxygenase and is 100-1,000 times faster than those found in eukaryotic systems [54, 55].

Changing the regioselectivity of hydroxylation to the ω-5 and ω-6 positions would enable the production of 4- and 5-hydryoxydecanoic acid required for decalactone formation (Fig. 1b). Regioselectivity altering mutations at S72Y, V78, F87 and I263 residues have previously been reported, with hydroxylation up to the ω-9 position on dodecanoic acid achieved, though this was accompanied by a loss of overall activity [57]. Mutations that increase BM3’s activity on fatty acids have also been reported in the literature, with different mutations displaying increased activity on C8–C16 fatty acids [58–61]. Mutants with altered substrate specificity have also been observed to have altered regioselectivities [58–60, 62], meaning combinatorial mutants with regioselectivityaltering and activity-enhancing mutations may not have the expected regioselectivities.

BM3 has previously been expressed in *E. coli* for the bioconversion of long chain fatty acids to the corresponding ω-1 to ω-3 hydroxyfatty acids [63], or incorporated into a synthetic metabolic pathway in *E. coli* with long chain acyl-ACP thioesterases to biosynthesise long chain ω-1 to ω-3 hydroxyfatty acids [64, 65].

## Materials and methods

### *E. coli* strains and culture conditions

*E. coli* DH5α (ThermoFisher Scientific) were used for molecular cloning of all plasmids produced during this work. *E. coli* JW17941 (BW25113 ΔfadD) was used for bioproduction and feeding experiments. The ΔfadD mutation prevents recycling of free fatty acids by the beta-oxidation pathway. [66] LB media was used to grow *E. coli* during cloning and to grow pre-cultures. M9 media was used to grow *E. coli* main cultures during bioproduction and feeding experiments. To make M9 media with bioavailable reduced iron (M9-Fe), M9 was supplemented with 6 mg/L Ferric Ammonium Citrate. Media were supplemented with antibiotics where appropriate.

### *E. coli* bioproduction and feeding experiments

The *E. coli* strains were first grown on LB-Agar. Three colonies were picked per plate and inoculated into 5 mL LB preculture with appropriate antibiotics in a 50 mL falcon tube. The precultures were incubated overnight at 37 °C, 180 rpm. The next day, the precultures were pelleted by centrifugation at 4000 rpm for 5 minutes. The supernatant was discarded, and the cells washed by resuspension in 2.5 mL M9. The precultures were centrifuged again at 4000 rpm for 5 minutes. The supernatant was discarded, and the pellets resuspended in 0.5 mL M9. The washed precultures were then used to inoculate 25 mL M9 with appropriate antibiotics in a 100 mL Erlenmeyer flask at an OD_600_ of 0.1. If an overlay was used, at this stage a 10% v/v overlay was added to M9. The cultures were then incubated at 37 °C, 150 rpm for 4 hrs, and then induced. If a feeding experiment was to be performed, the fed substrate was also added at this time. The cultures were then incubated at 30 °C, 150 rpm for 48 hrs, at which point samples were taken.

### *Synechocystis* Sp. PCC 6803 strains and culture conditions

*Synechocystis* Sp. PCC 6803 Δaas with Sfp-CAR integrated into the genome was used. This strain reduces free fatty acids to fatty alcohols, and the Δaas mutation prevents recycling of free fatty acids by the beta-oxidation pathway [67]. BG11 -Co media (BG11 media [68] with cobalt removed) was used for the cultivation of *Synechocystis*.

### *Synechocystis* bioproduction experiments

*Synechocystis* 6-well plate cultures were used to inoculate 25 mL BG11Co with appropriate antibiotics in a 100 mL Erlenmeyer flask at a starting OD_730_ of 0.2. Cultures were then incubated in an Algaetron AG230 at 30 °C, 180 rpm with continuous warm-white LED illumination at 50 μmol photons/m^2^ and a 1% CO_2_ atmosphere (v/v). After 2 days, the cultures were induced with cobalt (5 μM CO(NO_3_)_2_(H_2_O)_6_. Samples were then taken on Day 5 and Day 10.

### Plasmid assembly

Genes encoding *Cuphea viscossima* FatB1 (CvFatB1, GenBank: G3ESU9.1), BM3 (GenBank: WP_034650526.1), and *Umbellularia californica* FatB1 (UcFatB1, GenBank: Q41635.1) were codon-optimised for *Synechocystis* using IDTs codon optimisation tool and chemically synthesised as gBlocks by IDT. Genes were synthesised incorporating the BASIC [69] prefix and suffix sequences and were first cloned into pJET vectors. BASIC was then used to assemble the final expression vectors. Partially double stranded DNA linkers for BASIC were purchased from Biolegio. Linker sequences are listed in Supplementary Table 1.

### CvFatB1 truncation

CvFatB1 was truncated by PCR using Q5 DNA polymerase. The pJET containing the wild-type thioesterase was used as the template for the PCR reaction. The variable forwards primer bound at the site where the truncation was to be introduced and amplified the thioesterase from this point, adding the BASIC prefix and if necessary, a start codon. Meanwhile the reverse primer was kept constant and amplified the thioesterase while adding the BASIC suffix. The result was a N-terminally truncated thioesterase with BASIC prefix and suffix. Primers are listed in Supplementary Table 2.

### Gas-Chromatography MassSpectrometry (GC-MS)

GC-MS was used for the detection and quantification of volatile products. Samples were analysed using an Agilent 7890B GC with an Agilent DBWAXetr column and an Agilent 7693 autosampler, connected to an Agilent 5977B Mass Selective Detector. Samples were injected in PulsedSplit mode in a 3:1 ratio with a flow rate of 1.5 mL/min. The inlet temperature was 250 °C and inlet pressure was 10.3 psi. The carrier gas helium at a flow rate of 1.5 mL/min. The autosampler needler was washed with acetone and hexane before injecting samples. Acetone samples were run before samples to clean the column from any residue from previous experiments.

### Extraction of fatty alcohols in the presence of an isopropyl myristate overlay

A 10% v/v isopropyl myristate overlay was used to extract fatty alcohols from the culture media during bioproduction experiments. After the cultures had grown for 48 hrs the overlay developed a frothy appearance due to emulsion of the overlay and culture media. 1 mL of this emulsion was transferred to an Eppendorf tube and centrifuged at 13000 rpm for 1 min. After centrifugation 100 μL of the top layer (the overlay) was transferred to a 2 mL glass screwcap vial (Agilent) with a 250 μL insert (Agilent). Samples were then capped with a blue screw cap (Agilent).

Samples were injected into the GC-MS as describe above and then run with the following program. The oven temperature was initially set at 100 °C and held for 30 s. The temperature was then ramped at a rate of 30 °C /min to a final temperature of 250 °C, where it was held for 1 min.

### Extraction of decanoic acid and decalactones from the culture media

Chloroform-methanol was used to extract decanoic acid and decalactones from the cell cultures during feeding and bioproduction experiments. After 48 hrs cultivation 1 mL of cell culture was transferred to a capped glass tube. If the cell and aqueous phase were to be separately examined 1 mL cell culture was transferred to a 1.5 mL Eppendorf and centrifuged at 13000rpm for 10 mins. The supernatant was then transferred to a capped glass tube. The pellet was resuspended in 1 mL fresh media and transferred to a capped glass tube. 2 mL of 2:1 chloroform-methanol was added to the glass tube. If the sample was to be acidified 150 μL of 6 M HCl was also added to the glass tube, if not this step was skipped. The glass tube was vortexed and then centrifuged at 3500 rpm for 3 mins. After centrifugation, the bottom phase (chloroform) was transferred to a new capped glass tube using a glass Pasteur pipette. Samples were then dried under a nitrogen gas flow. Once dry, 100 μL of chloroform was added to the glass tube, and the glass tube was vortexed again. The chloroform was then transferred using a glass Pasteur pipette to a 2 mL glass screwcap vial with a 250 μL insert. Samples were then capped with a blue screw cap.

Samples were injected into the GC-MS as describe above and then run with the following program. The oven temperature was initially set at 160 °C and held for 30 s. The temperature was then ramped at a rate of 50 °C /min to 200 °C, where it was held for 2 mins. The temperature was then ramped again at 50 °C /min to a final temperature of 250 °C, where it was held for 1 min. 3.10 Prediction of the chloroplast localisation peptide

The chloroplast localisation peptide was predicted using the ChloroP server (http://www.cbs.dtu.dk/services/ChloroP/) [70]. At the time of writing this server has now been discontinued.

### Generation of predicted protein structures and structure guided mutagenesis

Predicted protein structures were generated using the Phyre2 server (http://www.sbg.bio.ic.ac.uk/%7Ephyre2/html/page.cgi?id=index) [71]. The amino acid sequence was inputted, and normal modelling mode was used. To generate multiple predicted protein structures at once Batch Processing mode was used. The Mutagenesis Wizard feature was used to investigate potential mutations that would cause steric hindrance at the appropriate location on the bound ligand.

### Statistical analysis

A Welch T-test was performed using R Studio. A *p*value of < 0.05 was taken to be significant, and all *p*values are displayed to 3 significant figures. Data presented is the average of 3 biological replicates, and error bars of ±1 Standard Deviation are shown.

## Results

### Truncation of *cuphea viscosissima* FatB1

CvFatB1 was chosen as candidate for engineering a C10 thioesterase, as it had the highest native C10 specificity [27]. To generate an active version of CvFatB1, the first step was to introduce an appropriate N-terminal truncation.

A set of systematic truncations was designed and produced. The truncations were designed using three approaches. First, CvFatB1 was truncated to excise the chloroplast localisation peptide predicted by the ChloroP server. Second, CvFatB1 was truncated according to previously reported truncations of FatB thioesterases in the literature [28, 30, 44, 48, 50, 51, 72, 73]. The basis upon which most of these previously reported truncation locations had been chosen was unclear. Finally, to cover for the possibility that the optimal truncation site was located elsewhere, CvFatB1 was systematically truncated every 10 amino acids, extending from the site of greatest truncation reported in the literature. In total this process generated 21 unique truncations of CvFatB1, some of which had already been produced in prior work by the Jones lab (Supplementary Table 3). These truncations were named CvT1-21, in order of truncation size, with T1 having the shortest truncation and T21 the longest.

**Fig. 2:**
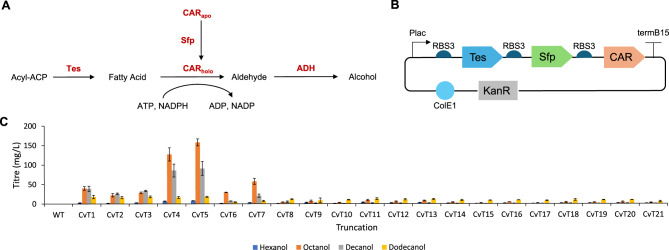
Screening CvFatB1 truncations **A**) Synthetic metabolic pathway for alcohol biosynthesis. Pathway enzymes are highlighted in red. Tes: CvFatB1 truncation, Sfp: Phosphopantetheine transferase (*Bacillus subtilis*), CAR: Carboxylic acid reductase (*Mycobacterium marinum*), ADH: Native alcohol dehydrogenases **B**) Plasmid design for expressing the alcohol biosynthesis pathway. A pET plasmid with ColE1 origin of replication and kanamycin resistance cassette was used to express pathway enzymes. Plac and RBS3 were used to express a CvFatB1 truncation, Sfp and CAR in a single operon. **C**) Alcohol titres after 48 hrs cultivation in M9 media with 2% glucose and a 10% v/v isopropyl myristate overlay. The overlay was sampled and analysed by GCMS. Cells were induced with 0.5 mM IPTG. Data is the average of 3 biological replicates and error bars are of 1 standard deviation

To screen the CvFatB1 truncations, a previously reported alcohol biosynthesis pathway was used [74] (Fig 2A). All heterologous genes in the pathway were expressed on a single plasmid (Fig 2B). By screening for alcohol biosynthesis, rather than acid biosynthesis, product analysis was simpler and faster, allowing for a larger set of truncations to be screened.

No alcohol biosynthesis was observed when expressing the native CvFatB1 wild-type (WT), while the highest alcohol titres were observed with truncations CvT4 and CvT5 (Fig 2C). The majority of alcohols produced after expression of CvT4 and CvT5 were octanol (127.1 mg/L and 157.7 mg/L, respectively) and decanol (85.5 mg/L and 90.8 mg/L, respectively). From CvT8 onwards, there was a loss of activity with only low titres of alcohols produced.

**Fig. 3:**
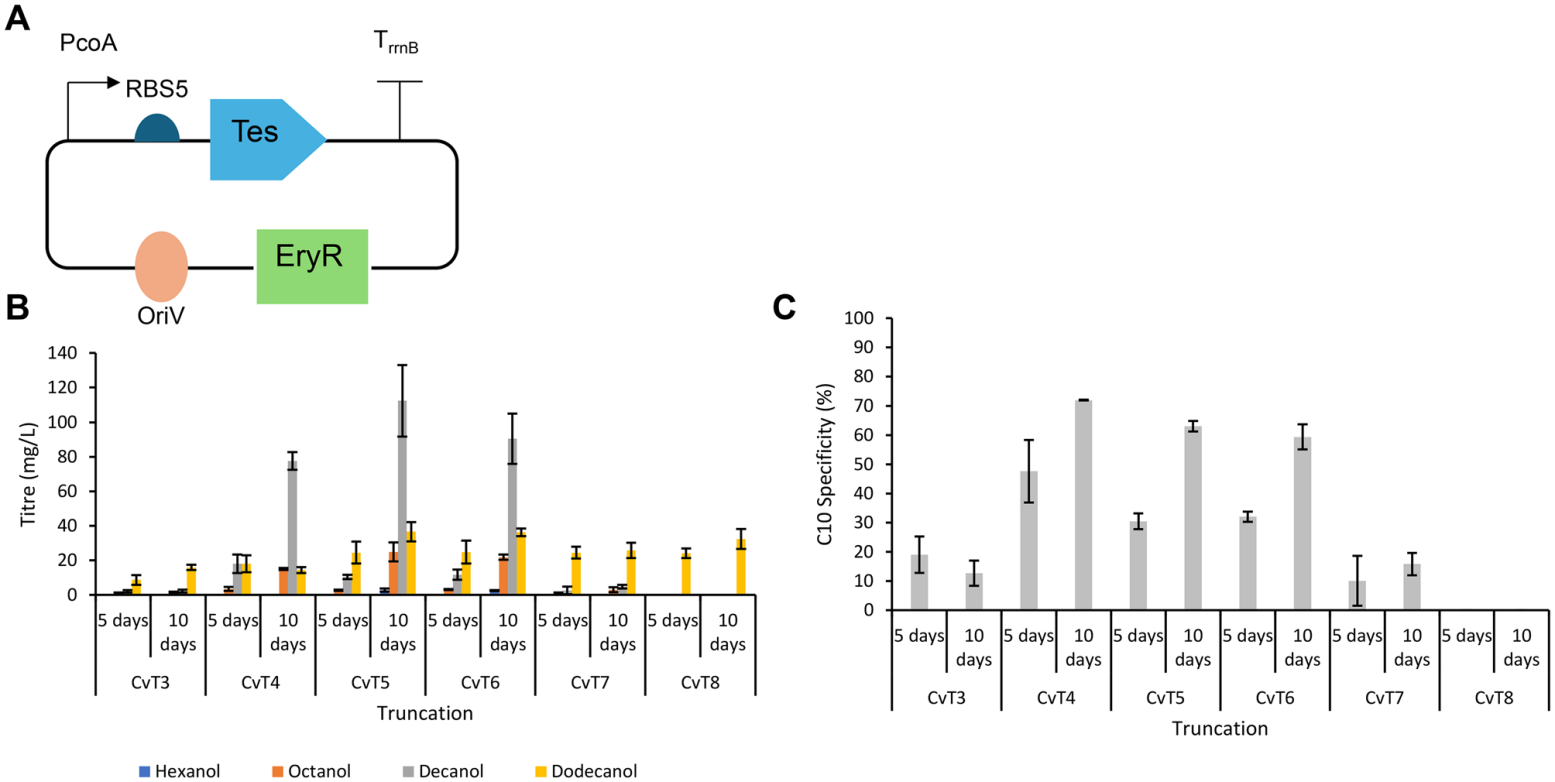
Expressing CvFatB1 truncations in *Synechocystis* **A**) a pRSF1010 plasmid with erythromycin resistance cassette was used to express a truncation of CvFatB1 (tes). The truncation was expressed using the PcoA promoter and RBS5. **B**) alcohol titres after 5 and 10 days cultivation in BG11-co media with a 30% v/v isopropyl myristate overlay. The overlay was sampled and analysed by GC-MS. Cells were induced with 5 μM cobalt. **C**) C10 specificity after 5 and 10 days cultivation, calculated as the percentage of total moles of fatty alcohol that were decanol. Data is the average of 3 biological replicates and error bars are of 1 standard deviation

CvT3–T8 were also screened in the cyanobacteria *Synechocystis* Sp. PCC 6803 Δaas with Sfp and CAR integrated into the genome [67]. Truncated thioesterase variants were expressed using a plasmid under the control of the PcoA promoter (Fig. 3A), with the truncations CvT4-6 resulting in the highest alcohol titres. In contrast to the results with *E. coli,* more decanol was produced than octanol. For example, strains expressing CvT5 accumulated higher decanol (112.4 mg/L) than octanol (24.9 mg/L) after 10 days of cultivation (Fig. 3B). CvT4 showed the highest C10 specificity (71.9%) compared to CvT5 (63.1%, *p* = 0.0135) and CvT6 (59.4%, *p* = 0.0376). There was no significant difference in the C10 specificity between CvT5 and CvT6 (*p* = 0.276) (Fig. 3C).

Following this observation, it was hypothesised that the differences in apparent thioesterase specificity following expression in *E. coli* and *Synechocystis* was due to the interactions between the thioesterases and the different ACPs. This theory was tested using CvT5. Expression of CvT5 was first inducer optimised (Supplementary Figure 1), and then overexpression of ACP from *Synechocystis* and *Cuphea lanceolata* in *E. coli* as a method of tailoring thioesterase specificity was investigated but had no effect on specificity. However, it was observed that overexpression of *E. coli* ACP, used as a control, led to a significant increase in titres, and so overexpression of EcACP was used going forward (Supplementary Figure 2).

### Site-directed mutagenesis of CvT5

**Fig. 4:**
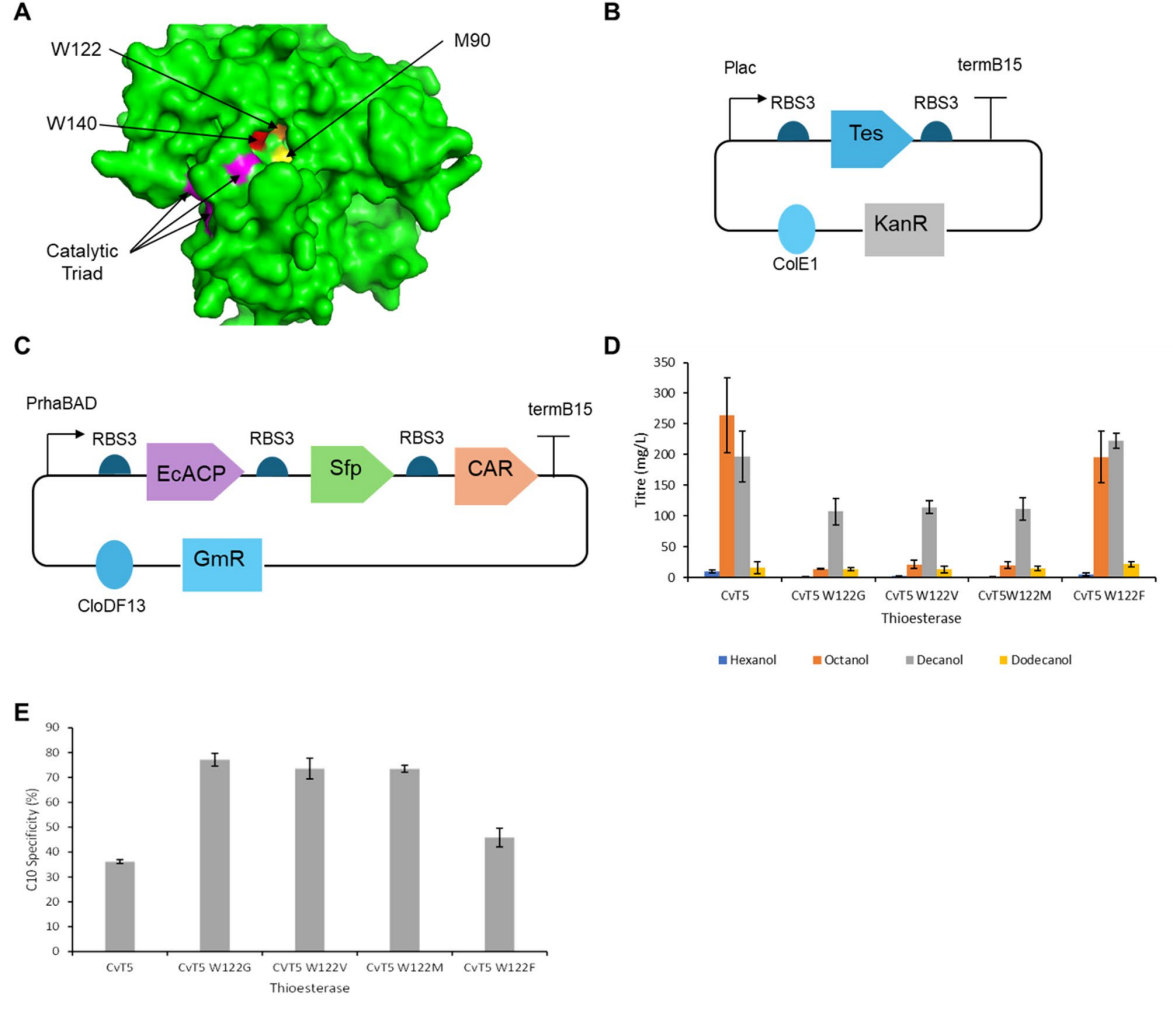
Engineering CvT5 **A**) the protein structure of CvT5 was predicted using Phyre2 and visualised using PyMol. The catalytic triad was identified (purple). Three large hydrophobic amino acid residues lining the bottom of the substrate binding pocket were identified; M90 (yellow), W122 (orange) and W140 (red). **B**) screening CvT5 122W mutants. A pET plasmid with kanamycin resistance cassette was used to express an engineered version of CvT5 (tes). Engineered thioesterases were expressed using the plac promoter and RBS3 **C**) a pCDF plasmid with gentamycin resistance cassette was used. EcACP, Sfp and car were expressed in a single operon using PrhaBAD and RBS3. E. coli ΔfadD were cotransformed with two plasmids for alcohol biosynthesis, expressing a CvT5 or a CvT5 W122 mutant and EcACP, Sfp and car. **D**) alcohol titres after 48 hrs cultivation in M9 media with 2% glucose and a 10% v/v isopropyl myristate overlay. The overlay was sampled and analysed by GC-MS. Cells were induced with a variable amount of IPTG and 1% rhamnose and the following concentrations of IPTG for each thioesterase: CvT5–75 μM, CvT5 W122G 250 μM, CvT5 W122V − 500 μM, CvT5 W122M − 500 μM, CvT5 W122F − 75 μM. **E**) C10 specificity, calculated as the percentage of total moles of fatty alcohol that were decanol. Data is the average of 3 biological replicates and error bars are of 1 standard deviation

As the thioesterase CvT5 resulted in the highest alcohol titres in *E. coli*, this variant was selected for further engineering. A model of CvT5’s structure was generated using the Phyre2 server [71] and visualised using PyMol. The catalytic triad was identified by alignments of the amino acid sequences of CvFatB1 and the *Umbellularia California* FatB (UcFatB) thioesterase, whose catalytic triad had previously been reported [51]. Upon examination of the protein structure, an internal cavity whose mouth was flanked by the catalytic triad was observed. This cavity was assumed to be the substrate binding pocket. Upon inspection of the cavity, three large hydrophobic residues, M90, W122 and W140 were identified that lined the bottom of the cavity (Fig. 4A). These were mutated to glycine, with the aim of enlarging the substrate binding pocket, and therefore increase CvT5’s C10 specificity. This approach had previously been reported to shift the specificity of UcFatB to favour longer chain length fatty acids [51].

The mutated CvT5 variants were screened by expression in *E. coli* (Fig. 4B), in combination with overexpression of EcACP, and expression of Sfp and CAR (Fig. 4C). As it had previously been reported that engineering thioesterases to alter the specificity resulted in an activity loss [50, 51], inducer optimisation was performed for each mutant in case the optimum IPTG concentration had shifted from that of the original CvT5 thioesterase. The M90G and W140G mutations led to severe losses of activity. The W122G mutation led to increased C10 specificity but also decreased alcohol titres (Supplementary Fig. 3). It was theorised that mutagenesis of W122 to a differently sized hydrophobic amino acid residue than glycine could produce a mutant of CvT5 that had increased C10 specificity without such a large activity loss. The following mutants; W122V, W122M and W122F were also generated, and screened with inducer optimisation as before (Supplementary Fig. 3). CvT5, CvT5 W122G, CvT5 W122V, CvT5 W122M and CvT5 W122F were then expressed using the optimum IPTG concentration for each thioesterase. The CvT5 W122V and CvT5 W122M mutants showed similar activity profiles to CvT5 W122G, with reduced alcohol titres, and with decanol being the most produced alcohol. The CvT5 W122F mutant showed a similar overall activity profile to CvT5, but with slightly more decanol produced than octanol. The highest decanol titres (222.5 mg/L) were observed following expression of CvT5 W122F (Fig. 4D) while the highest C10 specificity (77.1%) was observed following expression of CvT5 W122G (Fig. 4E).

### Mutagenesis of BM3

During initial experiments to test experimental protocols it was observed that feeding *E. coli* expressing wild-type BM3 with methyl decanoate led to the production of small amounts of decalactones. After feeding methyl decanoate decanoic acid was obtained, suggesting that the fed ester had been hydrolysed. Feeding methyl decanoate to *E. coli* expressing CYP450 from *Tepidophilus thermophilus* (TtCYP450), a catalytically self-sufficient CYP40 had been reported to hydroxylate decanoic acid with 90% ω5 regioselectivity [75], or an uncharacterised CYP450 from *Rhodocyclales bacterium* (RbCYP450) found by BlastP search that had 92.8% sequence similarity to TtCYP450, led to production of only trace amounts of δ-decalactone (Supplementary Figure 4). Therefore, it was decided to focus engineering efforts on BM3 with the aim to obtain a mutant with increased decalactone titres.

Firstly, BM3 was engineered to add either the single V78A mutation or the double V78A F87A mutation, as these changes had previously been reported to alter the regioselectivity of BM3 [57]. These BM3 mutants were then further engineered by also adding an L188Q [60] mutation, which had previously been reported to increased activity, or L188Q and I401P [76], another mutation that had previously been reported to increase activity.

**Fig. 5:**
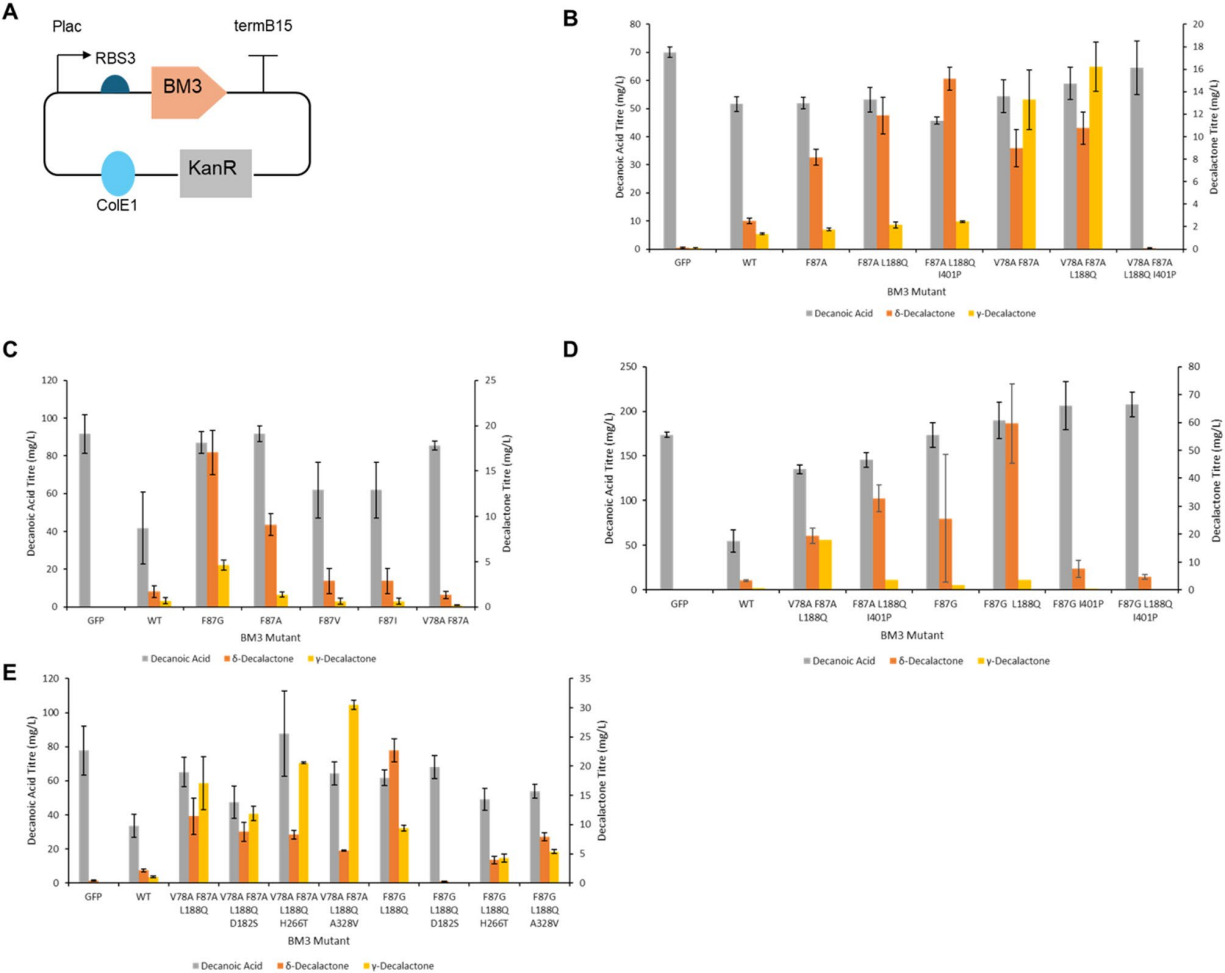
Screening BM3 mutants *E. coli* ΔfadD were transformed with a plasmid expressing either GFP, BM3 WT or a BM3 mutant. A) Plasmid design for expressing BM3. A pET plasmid with ColE1 origin of replication and kanamycin resistance cassette was used. Plac and RBS3 were used to express GFP, BM3 or a BM3 mutant B, C, D & E) Decanoic acid, δ-decalactone and γ-decalactone titres after cultivation in M9-Fe media for 48 hrs at 30°C, 150rpm. Cells were induced with 0.5 mM IPTG and fed with 2.5 mM methyl decanoate. Products were extracted from the media using chloroform methanol and sulfuric acid and analysed by GC-MS. Data is the average of 3 biological replicates and error bars are of 1 standard deviation.

All strains expressing the mutated variants of BM3 mutants resulted in cultures accumulating higher decanoic acid titres higher than in cultures of strains expressing the wild-type BM3 (BM3 WT). The BM3 F87A mutant produced significantly more δ-decalactone (8.17 mg/L, *p* = 0.00233) than the BM3 WT. Addition of the L188Q mutation increased the δ-decalactone titre to 11.8 mg/L, and addition of the L188Q and I401P mutations further increased the titre to 15.2 mg/L, significantly higher than BM3 F87A titres (*p* = 0.000997). The BM3 V78A F87A mutant produced significantly more δ-decalactone (8.96 mg/L, *p* = 0.0193) and γ-decalactone (13.2 mg/L, *p* = 0.0160) than the BM3 WT. Addition of the L188Q mutation increased both δ-decalactone and γ-decalactone titres to 10.8 mg/L and 16.2 mg/L, but this increase was not significant. Further addition of the I401P mutation abolished γ-decalactone production, leaving only trace amounts of δ-decalactone (Fig. 5B).

Mutagenesis of the F87 site was then further explored, as different point mutations at this site had been reported to have different regioselectivities to F87A. F87G and F87V mutations from the literature [57], as well as a novel F87I mutation were screened by a feeding assay. GFP, BM3 WT, BM3 F88A and BM3 V78A F87A were used as controls. Decanoic acid titres were similar in *E. coli* expressing BM3 F87G and F87A, while F87V and F87I had reduced decanoic acid titres relative to F87A. The BM3 F87G mutant had significantly higher δ-decalactone titres (17.0 mg/L, *p* = 0.0160) and γ-decalactone titres (4.63 mg/L, *p* = 0.00302) than the BM3 F87A mutant. δ-decalactone and γ-decalactone titres in the BM3 V78A F87A mutant were lower than previously observed, making comparison of BM3 F87G and BM3 V78A F87A difficult. The F87V and F87I mutants had slightly higher δ-decalactone titres than the BM3 WT, but titres were lower than those of the original F87A mutant (Fig. 5C).

Most of the fed substrate was not consumed, therefore addition of the activity enhancing mutations L188Q and I401P to BM3 F87G was then explored. Combinatorial mutants were generated and screened using a feeding experiment. *E. coli* expressing GFP, BM3 WT, BM3 V78A F87A L188Q and BM3 F87A L188Q I401P were used as controls. It was noticed that titres of decanoic acid, δ-decalactone and γ-decalactone were higher than normally observed in the controls. Despite this, inferences could be made about the relative activities of the new mutants. Combining activity enhancing mutations with F87G unexpectedly led to higher decanoic titres, although the increases in titre were not significant. Only the BM3 F87G L188Q mutant showed increased δ-decalactone titres, which reached 59.7 mg/L. This was an increase relative to both BM3 F87G (25.7 mg/L) and BM3 F87A L188Q I401P (32.8 mg/L), however due to large variation observed for all the mutants these increases were not significant. The I401P mutation appeared detrimental to activity, with both the BM3 F87G I401P and BM3 F87G L188Q I401P mutants producing less δ-decalactone than BM3 F87G (Fig. 5D).

Further activity enhancing mutations D182S and H266T [77], and A328V [59] were added to the BM3 V78A F87A L188Q and BM3 F87G L188Q combinatorial mutants. Incorporating the D182S, H266T and A328V point mutations had no effect on decanoic acid titres. When D182S, H226T or A328V were combined with BM3 F87G L188Q titres of both δ-decalactone and γ-decalactone fell significantly. On the other hand, combining D182S and H266T with BM3 V78A F87A L188Q had no significant effect on δ-decalactone and γ-decalactone titres. Combination of A328V with BM3 V78A F87A L188Q led to significantly higher γ-decalactone titres (30.5 mg/L, *p* = 0.0327) and an insignificant decrease in δ-decalactone titres (5.53 mg/L, *p* = 0.0806) (Fig. 5E).

### *De novo* biosynthesis of decalactones

**Fig. 6:**
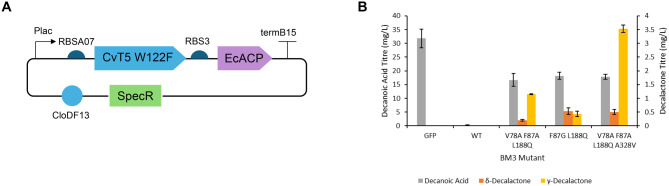
Biosynthesis of decalactones *E. coli* ΔfadD were transformed with a plasmid expressing EcACP and CvT5 W122F and a second plasmid expressing GFP or a BM3 mutant. **A**) plasmid design for expressing CvT5 W122F and EcACP. A pCDF plasmid with CloDF13 origin of replication and kanamycin resistance cassette was used. Plac was used to express both genes in a single operon **B**) Decanoic acid, δ-decalactone and γ-decalactone titres after cultivation in M9-Fe media for 48 hrs at 30 °C. Cells were induced with 0.5 mM IPTG. Products were extracted from the media using chloroform-methanol and sulfuric acid and analysed by GC-MS. Data is the average of 3 biological replicates and error bars are of 1 standard deviation

Following the search for and the engineering of suitable enzymes, the final aim was to complete a synthetic metabolic pathway for biosynthesis of decalactones in vivo. The CvT5 W122F thioesterase was selected for biosynthesis of decanoic acid, as its expression had previously led to the highest decanol titres. As BM3 had been reported to have very low activity on octanoic acid [61], off-target activity of BM3 on octanoic acid was likely to not be an issue. Therefore, it was decided that using a thioesterase that produced more decanoic acid over a thioesterase that specifically produced decanoic acid would lead to the highest decalactone titres. EcACP and CvT5 W122F were therefore co-expressed using one plasmid, with a slightly weaker RBS used to express CvT5 W122F (Fig. 6A), whilst the BM3 mutants were expressed using the same plasmids as before.

Decanoic acid titres of 31.8 mg/L were observed when GFP was expressed on the second plasmid instead of a BM3 mutant. This was substantially lower than the decanol titres that had previously been obtained. The highest γ-decalactone titres (3.53 mg/L) were observed when the thioesterase was co-expressed with BM3 V78A F87A L188Q A328V. δ-decalactone titres were lower, reaching only 0.53 mg/L following expression of BM3 F87G L188Q (Fig. 6B). The BM3 V78A F87A L188Q and F87G L188Q mutants appeared to have altered regioselectivities compared to the feeding experiments, with BM3 V78A F87A L188Q shifting from producing roughly equal amounts of δ- and γ-decalactone to producing more γ-decalactone, and BM3 F87G L188Q shifting from producing more δ-decalactone to roughly equal amounts of δ- and γ-decalactone.

### *De novo* biosynthesis of dodecalactones

**Fig. 7:**
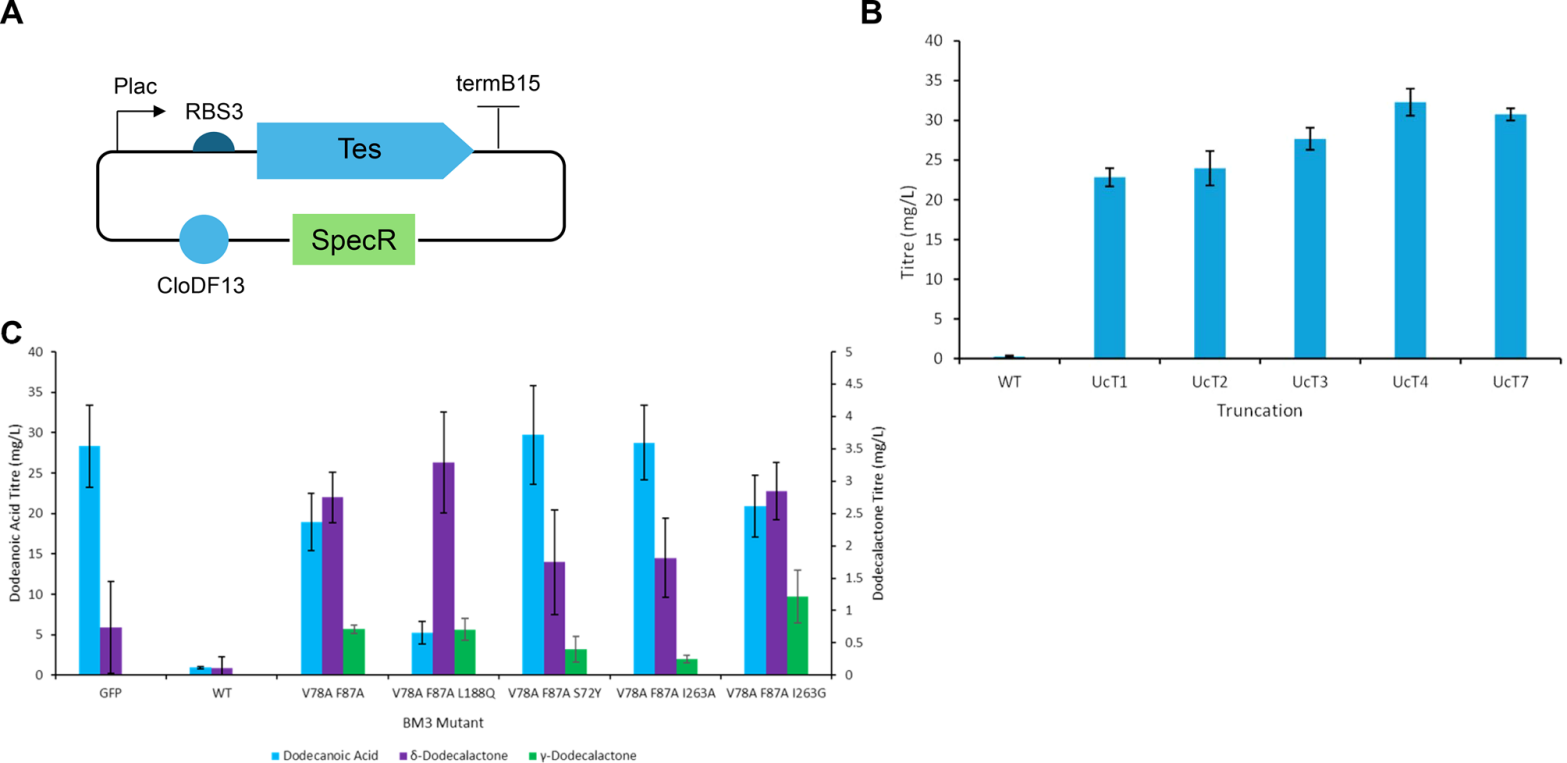
Biosynthesis of dodecalactones *E. coli* ΔfadD were transformed with a plasmid expressing a thioesterase and a second plasmid expressing a BM3 mutant. **A**) plasmid design for expressing thioesterase. A pCDF plasmid with CloDF13 origin of replication and spectinoymcin resistance cassette was used. Plac was used to express the thioesterase **B**) dodecanoic acid titres following expression of different UaFatB1 truncations without co-expression of BM3. **C**) dodecanoic acid, δ-dodecalactone and γ-dodecalactone titres after cultivation in M9Fe media for 48 hrs at 30 °C. Cells were induced with 0.5 mM IPTG. Products were extracted from the media using chloroform-methanol and sulfuric acid and analysed by GCMS. Data is the average of 3 biological replicates and error bars are of 1 standard deviation

Preliminary work was also performed to see if the pathway could be adapted to the biosynthesis of other medium chain-length γ- and δ-lactones, starting with dodecalactones. The FatB1 thioesterase from *Umbellularia californica* (UcFatB1) has been reported to be C12 specific [25], and so was chosen for the pathway. A small set of UcFatB1 truncations was generated and expressed using a plasmid (Fig. 7A) and screened in *E. coli*. Expression of UcT4 led to the highest dodecanoic acid titres (32.3 mg/L) (Fig. 7B) and so UcT4 was chosen for the pathway. A second plasmid was then used to co-express a selection of previously developed BM3 mutants and new mutants that were reported to have altered regioselectivity [57] with UcT4. Almost no decanoic acid was observed following co-expression of the BM3, and decanoic acid titres were significantly higher when either GFP or a BM3 mutant were co-expressed. The highest δ-dodecalactone titres (3.29 mg/L) were observed following expression of BM3 V78A F87V L188Q and the highest γ-dodecalactone titres (1.21 mg/L were observed following expression of BM3 V78A F87V I263G (Fig. 7C).

## Discussion

### Truncation of CvFatB1

In this work a much larger set of truncations was investigated during the truncation assay of CvFatB1 than had previously been examined in the literature when truncating other thioesterases.

From the truncation CvT8 onwards, there was a clear loss of activity with low alcohol titres observed, of which dodecanol followed by octanol were the major products. Truncation beyond the site of truncation used in CvT7 was likely detrimental to the thioesterase’s activity as both the N-terminal chloroplast localisation peptide and part of the thioesterase proper were removed. The highest alcohol titres were observed with CvT4–T7. This suggests that the optimal site for truncation of CvFatB1 is somewhere within the region between the truncations used to generate CvT4 and CvT7. The optimal truncation site of a FatB thioesterase has not previously been so precisely located, and it was not known that the residues between CvT7 and CvT8 played such an important, albeit still to be determined, role on thioesterase activity.

When the truncations CVT3-8 were expressed in *Synechocystis* a significant difference in specificity was observed, with more decanol than octanol being produced. The C10 specificity reached 71.9% when CvT4 was expressed which is the highest published C10 specificity observed for any acy-lACP thioesterase.

This increased C10 specificity when the thioesterase was heterologously expressed in *Synechocystis* matched previous observations from the Jones lab where a thioesterase, *Cuphea hookerina* FatB2, that had mixed C8 and C10 specificity upon expression in *Synechocystis* [49] was then found in unpublished work to be C8 specific when expressed in *E. coli*.

A potential explanation is that there could be differences in the fatty acid biosynthetic cycle between *Synechocystis* and *E. coli* that leads to a relatively larger amount of decanoyl-ACP being present in *Synechocystis*. This increased substrate availability would therefore lead to more decanoic acid and therefore decanol being biosynthesised, explaining the higher apparent C10 specificities.

### Engineering CvT5 for increased C10 specificity in *E. coli*

Structure guided mutagenesis of CvT5 led to the following three mutants being designed and tested, CvT5 M90G, CvT5 W122G and CvT5 W140G. Of these, CvT5 M90G and CvT5 W140G displayed a loss of activity, suggesting mutation at these sites were detrimental to the thioesterase activity. M90 and W140 line the putative substrate binding pocket, and their mutation to glycine could have destabilised the protein structure, leading to the observed activity loss.

On the other hand, CvT5 W122G displayed increased C10 specificity, which reached as high as 77.1% (Fig. 4E), but at a cost to the overall activity, resulting lower product titres. After this work had been completed, an engineered version of *Cuphea lanceolata* FatB3 (ClFatB3) that incorporated a truncation and activity enhancing mutations was reported to have 70% C10 specificity. While this C10 specificity was lower than that of CvT5 W122G, this was achieved without the need for specificity-altering mutations [46]. The application of the engineering techniques used on CvT5 to the engineered ClFatB3 could therefore result in even more specific C10 acylACP thioesterases in future work.

After optimising the mutation at the W122 position, the CvT5 W122F mutant was obtained which also showed slightly increased C10 specificity (46.9%), this time without losing any activity, and was used to biosynthesise lactones due to its ability to produce more decanoic acid compared to the other mutants.

### The regioselectivity and substrate specificity of BM3

It was observed that feeding decanoic to the BM3 WT resulted in low amounts of δ-decalactone and γ-decalactone being produced. In previous work characterising the regioselectivity of BM3 and BM3 mutants when hydroxylating dodecanoic acid, hydroxylation beyond the ω-4 position was not observed [57]. However, to produce δ-decalactone and γ-decalactone, ω-5 and ω-6 hydroxylation is necessary, meaning that BM3 has a much wider native regioselectivity on decanoic acid. This is the first time that hydroxylation at these positions by nonengineered BM3 has been reported.

While BM3 was able to convert some of the decanoic acid, the majority was not hydroxylated. Hydroxylation of decanoic acid by BM3 has previously been reported, but BM3 is more active on longer chain length substrates [60, 61]. Adding the activity enhancing mutation L188Q did increase decanoic acid consumption, but regioselectivity altering mutations were observed to and have been reported to decrease BM3 activity. To efficiently hydroxylate decanoic acid at the ω-5 and ω-6 positions multiple activity enhancing mutations will likely be needed.

### Mutagenesis of BM3

It was observed that the regioselectivity altering mutations, for example F87A, had different apparent regioselectivities than what was previously reported [57]. These mutations were characterised using dodecanoic acid and so may have different regioselectivities on different chain length fatty acids. However, the mutations did also result in activity losses, as was reported in the literature [57].

Due to these activity losses, addition of different activity enhancing mutations from the literature were also tested [59, 60, 76, 77]. Three of them, L188Q, A328V and I401P were found to increase the activity of BM3 regioselectivity mutants. While L188Q could be combined with the different regioselectivity mutants, combining A328V and I401P with regioselectivity mutants sometimes increased decalactone titres, but sometimes they resulted in a decrease. This suggests that certain combinations of mutations may have been destabilising to the protein, resulting in a loss of activity. Alternatively, mutations altering fatty acid substrate specificity have also been reported to affect the regioselectivity [58–60, 62], and this could have resulted in the hydroxylation of decanoic acid at the incorrect positions for decalactone biosynthesis.

Two combinatorial mutants were obtained from the mutagenesis assays, BM3 F87G L188Q and BM3 V78A F87A L188Q A328V, that displayed the highest specificities for δ-decalactone and γ-decalactone, respectively. These mutants would allow tailoring of the proposed decalactone biosynthesis pathway to produce either the δ- or γ-decalactone.

### *De novo* Biosynthesis of decalactones

In this work *de novo* biosynthesis of both δ-decalactone and γ-decalactone was achieved in *E. coli*, achieving titres of 0.53 mg/L and 3.53 mg/L, respectively. While low, this result demonstrates that the novel metabolic pathway designed in this work is functional. Furthermore, *de novo* biosynthesis of lactones had not previously been reported in *E. coli*.

*De novo* biosynthesis of γ-lactones has been reported in *Ashbya gossypii*, with similarly low titres, reaching ~3 mg/L [15]. In that instance, a range of γlactones of different chain lengths were biosynthesised, whereas in this work γ- and δ-lactones of the same chain length were biosynthesised. In the former report, oleic acid was first biosynthesised and then fed into the β-oxidation cycle to produce shorter acylCoA and it is unclear how the shortened fatty acids were then hydroxylated and condensed to lactones [15].

The γ-decalactones titres achieved in this work were therefore similar to those previously reported. However, the system in this work is better understood as the biosynthesis and consumption of the decanoic acid could be observed, concurrent with the biosynthesis of decalactones. Furthermore, the output of the pathway could be tailored to production of a mixture of δ- and γ-decalactones, or to γ-decalactone. Two clear limiting factors for the pathway were identified: (1) lower than expected decanoic acid biosynthesis, and (2) incomplete utilisation of decanoic acid by the BM3 mutants.

Decanoic acid titres in the decalactone biosynthesis pathway were lower than expected compared to fatty alcohol biosynthesis when screening the mutant thioesterases. However, when the mutants were screened, they were co-expressed with Sfp and CAR, a highly active enzyme, and this could have led to increased flux if the resulting alcohols are more efficiently removed by native efflux transporters compared to the lactones. Furthermore, the hydrophobicity of the end-product, in combination with the isopropyl myristate overlay used when CvT5 W122F was characterised, likely influenced flux through the pathway as previously demonstrated [67].

While using the alcohol biosynthesis pathway as a method to screen thioesterase variants did increase the ease of screening, once leading variants had been identified they should have also been characterised individually, without co-expression of other enzymes that may affect the outcomes.

The other limiting factor was the poor conversion of decanoic acid to decalactones by BM3 mutants. When the decalactone biosynthesis pathway was expressed using CvT5 W122F and BM3 V78A F87A L188Q A328V, decanoic acid titres of 17.8 mg/L were observed, indicating that not all of the decanoic acid produced was hydroxylated. When CvT5 W122F and GFP were expressed, decanoic acid titres of 31.8 mg/L were obtained. It can be therefore estimated that approximately only 44.0% (14.0 mg/L) of the available decanoic acid substrate produced by CvT5 W122F was hydroxylated by the BM3 V78A F87A L188Q A328V.

Furthermore, δ-decalactone and γ-decalactone titres were 0.51 mg/L and 3.53 mg/L, respectively, indicating that only 3.6% and 25.2% of the decanoic acid hydroxylated by BM3 V78A F87A L188Q A328V was hydroxylated at the 5- and 4-positions, assuming full conversion of the hydroxyfatty acids to the respective lactones and no product loss. This means that the majority of hydroxylation performed by the BM3 mutant was off-target. There is therefore still a need for further work on engineering BM3 for increased activity and regioselectivity.

### Modifying the pathway for dodecalactone biosynthesis

Preliminary work was performed to see if the pathway could also be used to biosynthesise other medium-chain length γ- and δ-lactones, starting with dodecalactones. While only a relatively limited set of thioesterase truncations and BM3 mutants were tested, *de novo* biosynthesis of both γ-dodecalactone and δ-dodecalactone were observed. This demonstrates the versatility of the designed pathway, and how it can be tailored to produce different chain length γ- or δ-lactones.

## Electronic supplementary material

Below is the link to the electronic supplementary material.

Supplementary Material 1

## Data Availability

No datasets were generated or analysed during the current study.
