# Supplementary material for: A synthetic metabolic pathway for the de novo biosynthesis of medium chain length γ- and δ-lactones
Source: J Biol Eng. 2025 Nov 25;19:104. doi: 10.1186/s13036-025-00575-z (PMC12649083; doi:10.1186/s13036-025-00575-z)

## Slide 1
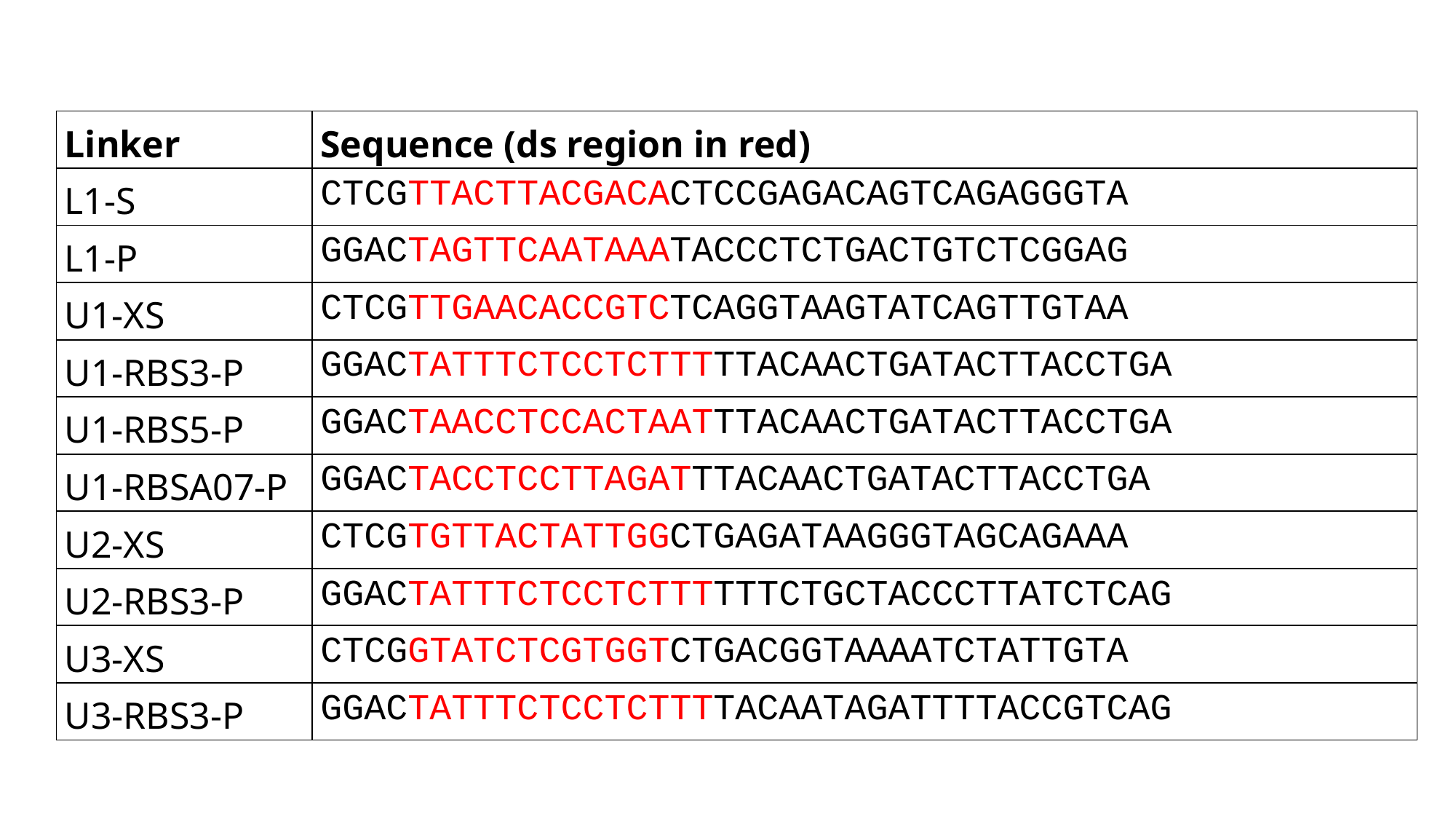

| Linker | Sequence (ds region in red) |
| --- | --- |
| L1-S | CTCGTTACTTACGACACTCCGAGACAGTCAGAGGGTA |
| L1-P | GGACTAGTTCAATAAATACCCTCTGACTGTCTCGGAG |
| U1-XS | CTCGTTGAACACCGTCTCAGGTAAGTATCAGTTGTAA |
| U1-RBS3-P | GGACTATTTCTCCTCTTTTTACAACTGATACTTACCTGA |
| U1-RBS5-P | GGACTAACCTCCACTAATTTACAACTGATACTTACCTGA |
| U1-RBSA07-P | GGACtaCCTCCTTAgatTTACAACTGATACTTACCTGA |
| U2-XS | CTCGTGTTACTATTGGCTGAGATAAGGGTAGCAGAAA |
| U2-RBS3-P | GGACTATTTCTCCTCTTTTTTCTGCTACCCTTATCTCAG |
| U3-XS | CTCGGTATCTCGTGGTCTGACGGTAAAATCTATTGTA |
| U3-RBS3-P | GGACTATTTCTCCTCTTTTACAATAGATTTTACCGTCAG |

## Slide 2
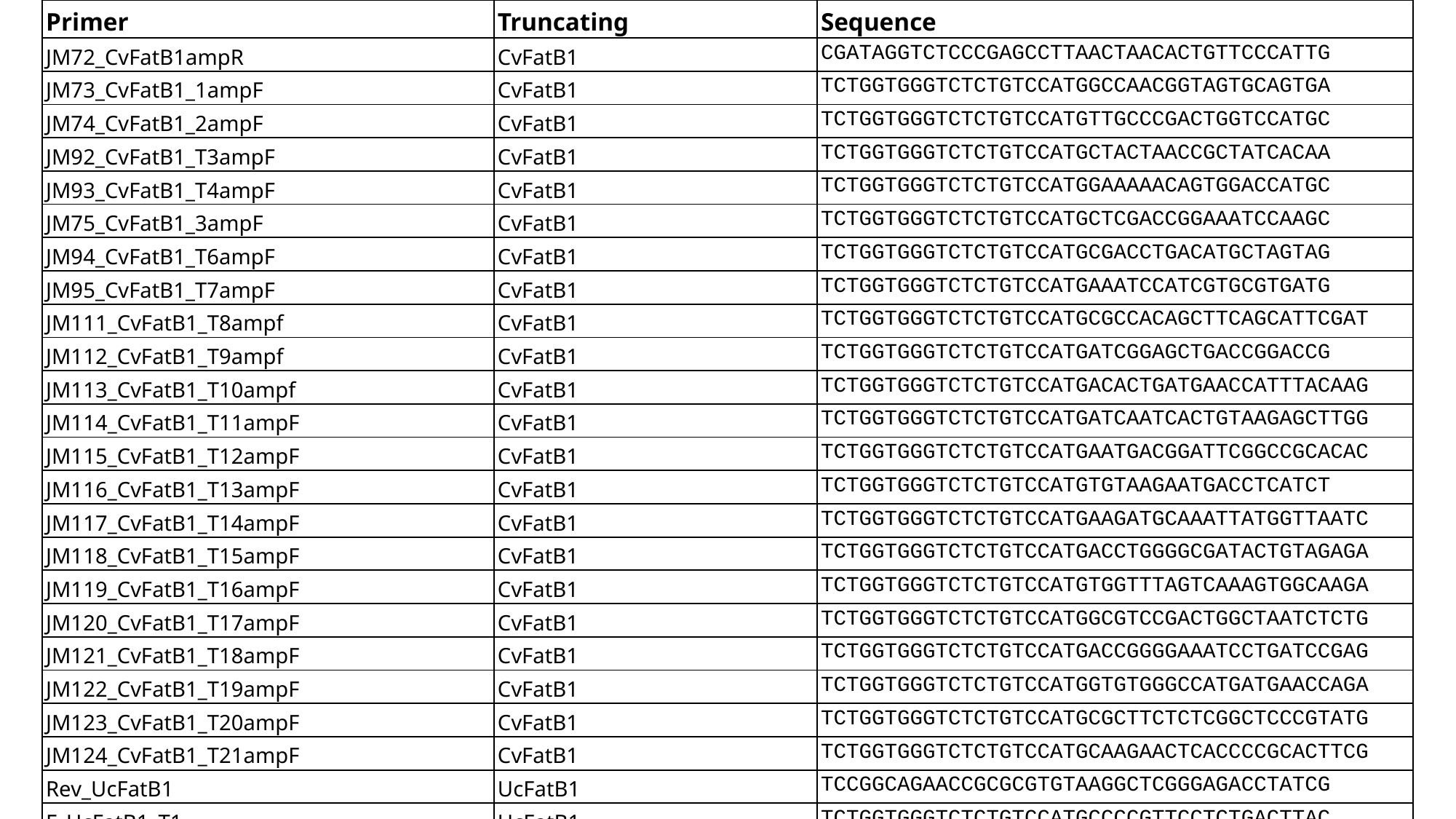

| Primer | Truncating | Sequence |
| --- | --- | --- |
| JM72\_CvFatB1ampR | CvFatB1 | CGATAGGTCTCCCGAGCCTTAACTAACACTGTTCCCATTG |
| JM73\_CvFatB1\_1ampF | CvFatB1 | TCTGGTGGGTCTCTGTCCATGGCCAACGGTAGTGCAGTGA |
| JM74\_CvFatB1\_2ampF | CvFatB1 | TCTGGTGGGTCTCTGTCCATGTTGCCCGACTGGTCCATGC |
| JM92\_CvFatB1\_T3ampF | CvFatB1 | TCTGGTGGGTCTCTGTCCATGCTACTAACCGCTATCACAA |
| JM93\_CvFatB1\_T4ampF | CvFatB1 | TCTGGTGGGTCTCTGTCCATGGAAAAACAGTGGACCATGC |
| JM75\_CvFatB1\_3ampF | CvFatB1 | TCTGGTGGGTCTCTGTCCATGCTCGACCGGAAATCCAAGC |
| JM94\_CvFatB1\_T6ampF | CvFatB1 | TCTGGTGGGTCTCTGTCCATGCGACCTGACATGCTAGTAG |
| JM95\_CvFatB1\_T7ampF | CvFatB1 | TCTGGTGGGTCTCTGTCCATGAAATCCATCGTGCGTGATG |
| JM111\_CvFatB1\_T8ampf | CvFatB1 | TCTGGTGGGTCTCTGTCCATGCGCCACAGCTTCAGCATTCGAT |
| JM112\_CvFatB1\_T9ampf | CvFatB1 | TCTGGTGGGTCTCTGTCCATGATCGGAGCTGACCGGACCG |
| JM113\_CvFatB1\_T10ampf | CvFatB1 | TCTGGTGGGTCTCTGTCCATGACACTGATGAACCATTTACAAG |
| JM114\_CvFatB1\_T11ampF | CvFatB1 | TCTGGTGGGTCTCTGTCCATGATCAATCACTGTAAGAGCTTGG |
| JM115\_CvFatB1\_T12ampF | CvFatB1 | TCTGGTGGGTCTCTGTCCATGAATGACGGATTCGGCCGCACAC |
| JM116\_CvFatB1\_T13ampF | CvFatB1 | TCTGGTGGGTCTCTGTCCATGTGTAAGAATGACCTCATCT |
| JM117\_CvFatB1\_T14ampF | CvFatB1 | TCTGGTGGGTCTCTGTCCATGAAGATGCAAATTATGGTTAATC |
| JM118\_CvFatB1\_T15ampF | CvFatB1 | TCTGGTGGGTCTCTGTCCATGACCTGGGGCGATACTGTAGAGA |
| JM119\_CvFatB1\_T16ampF | CvFatB1 | TCTGGTGGGTCTCTGTCCATGTGGTTTAGTCAAAGTGGCAAGA |
| JM120\_CvFatB1\_T17ampF | CvFatB1 | TCTGGTGGGTCTCTGTCCATGGCGTCCGACTGGCTAATCTCTG |
| JM121\_CvFatB1\_T18ampF | CvFatB1 | TCTGGTGGGTCTCTGTCCATGACCGGGGAAATCCTGATCCGAG |
| JM122\_CvFatB1\_T19ampF | CvFatB1 | TCTGGTGGGTCTCTGTCCATGGTGTGGGCCATGATGAACCAGA |
| JM123\_CvFatB1\_T20ampF | CvFatB1 | TCTGGTGGGTCTCTGTCCATGCGCTTCTCTCGGCTCCCGTATG |
| JM124\_CvFatB1\_T21ampF | CvFatB1 | TCTGGTGGGTCTCTGTCCATGCAAGAACTCACCCCGCACTTCG |
| Rev\_UcFatB1 | UcFatB1 | TCCGGCAGAACCGCGCGTGTAAGGCTCGGGAGACCTATCG |
| F\_UcFatB1\_T1 | UcFatB1 | TCTGGTGGGTCTCTGTCCATGCCCCGTTCCTCTGACTTAC |
| F\_UcFatB1\_T2 | UcFatB1 | TCTGGTGGGTCTCTGTCCATGCTTCCCGACTGGAGTATGTT |
| F\_UcFatB1\_T3 | UcFatB1 | TCTGGTGGGTCTCTGTCCATGATGTTATTTGCTGTGATTAC |
| F\_UcFatB1\_T4 | UcFatB1 | TCTGGTGGGTCTCTGTCCATGGAGAAACAGTGGACAAACTT |
| F\_UcFatB1\_T7 | UcFatB1 | TCTGGTGGGTCTCTGTCCATGCTTCACGGCCTTGTTTTCCG |

## Slide 3
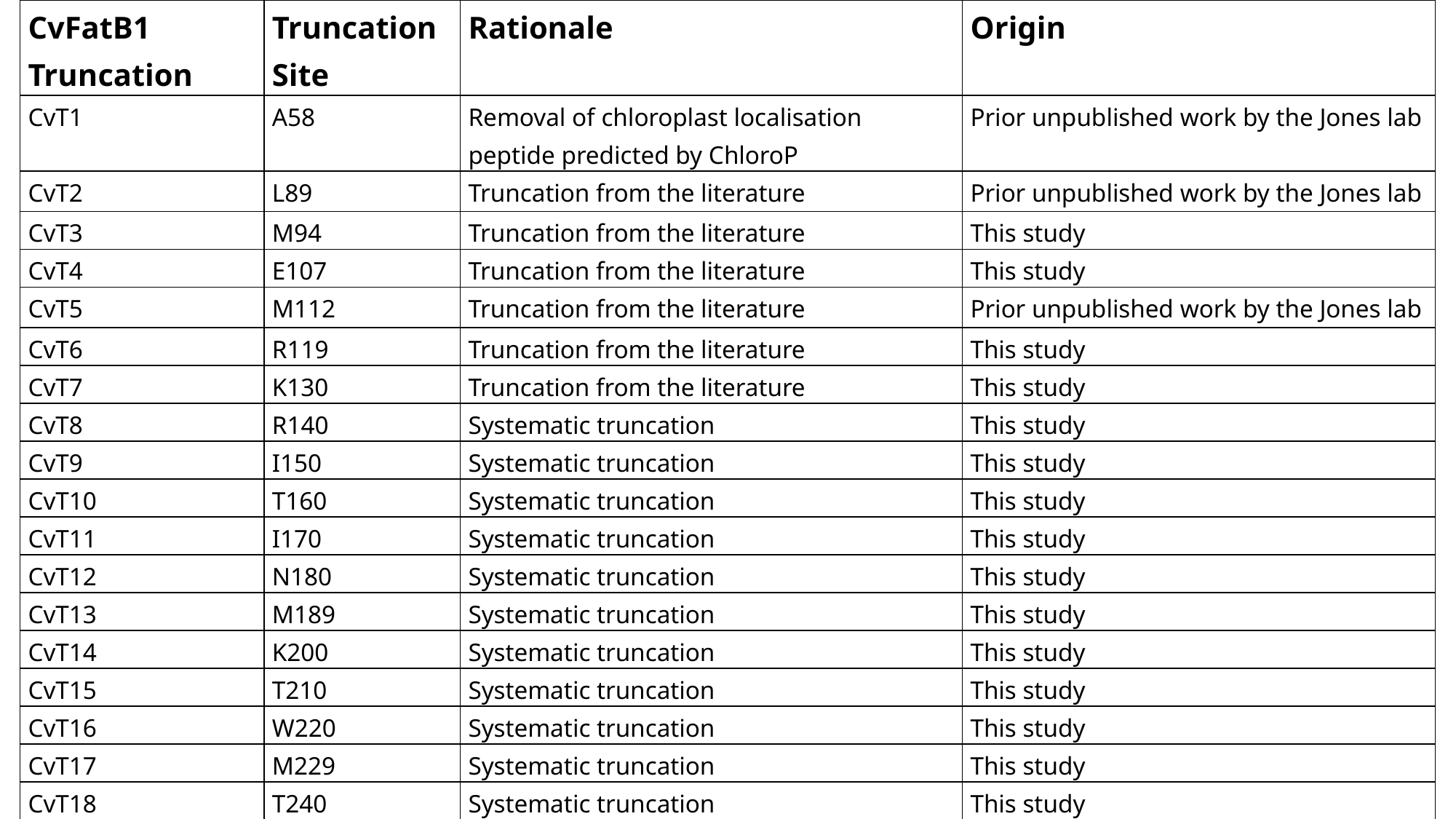

| CvFatB1 Truncation | Truncation Site | Rationale | Origin |
| --- | --- | --- | --- |
| CvT1 | A58 | Removal of chloroplast localisation peptide predicted by ChloroP | Prior unpublished work by the Jones lab |
| CvT2 | L89 | Truncation from the literature | Prior unpublished work by the Jones lab |
| CvT3 | M94 | Truncation from the literature | This study |
| CvT4 | E107 | Truncation from the literature | This study |
| CvT5 | M112 | Truncation from the literature | Prior unpublished work by the Jones lab |
| CvT6 | R119 | Truncation from the literature | This study |
| CvT7 | K130 | Truncation from the literature | This study |
| CvT8 | R140 | Systematic truncation | This study |
| CvT9 | I150 | Systematic truncation | This study |
| CvT10 | T160 | Systematic truncation | This study |
| CvT11 | I170 | Systematic truncation | This study |
| CvT12 | N180 | Systematic truncation | This study |
| CvT13 | M189 | Systematic truncation | This study |
| CvT14 | K200 | Systematic truncation | This study |
| CvT15 | T210 | Systematic truncation | This study |
| CvT16 | W220 | Systematic truncation | This study |
| CvT17 | M229 | Systematic truncation | This study |
| CvT18 | T240 | Systematic truncation | This study |
| CvT19 | V250 | Systematic truncation | This study |
| CvT20 | R260 | Systematic truncation | This study |
| CvT21 | Q270 | Systematic truncation | This study |

## Slide 4
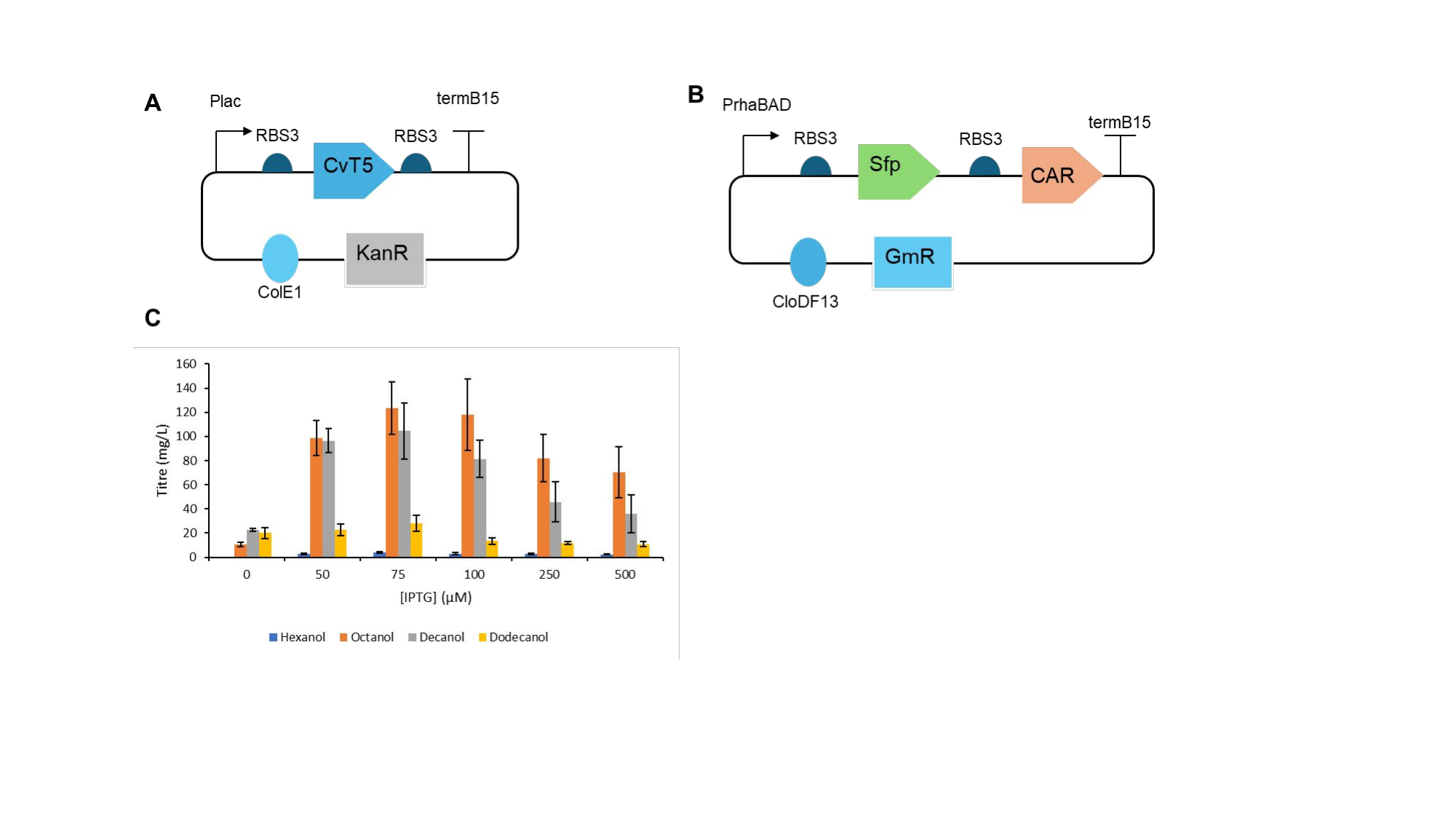

## Slide 5
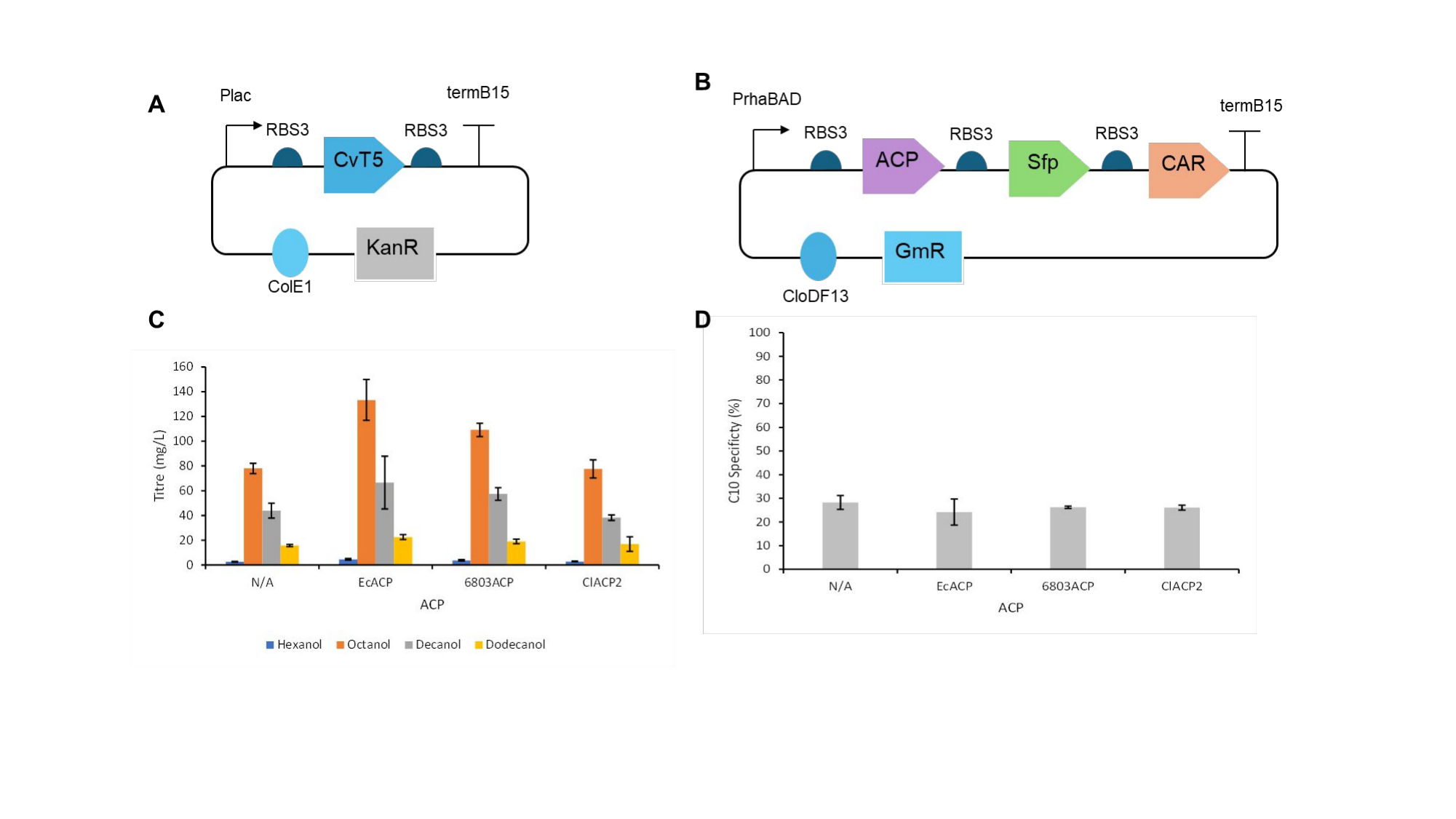

## Slide 6
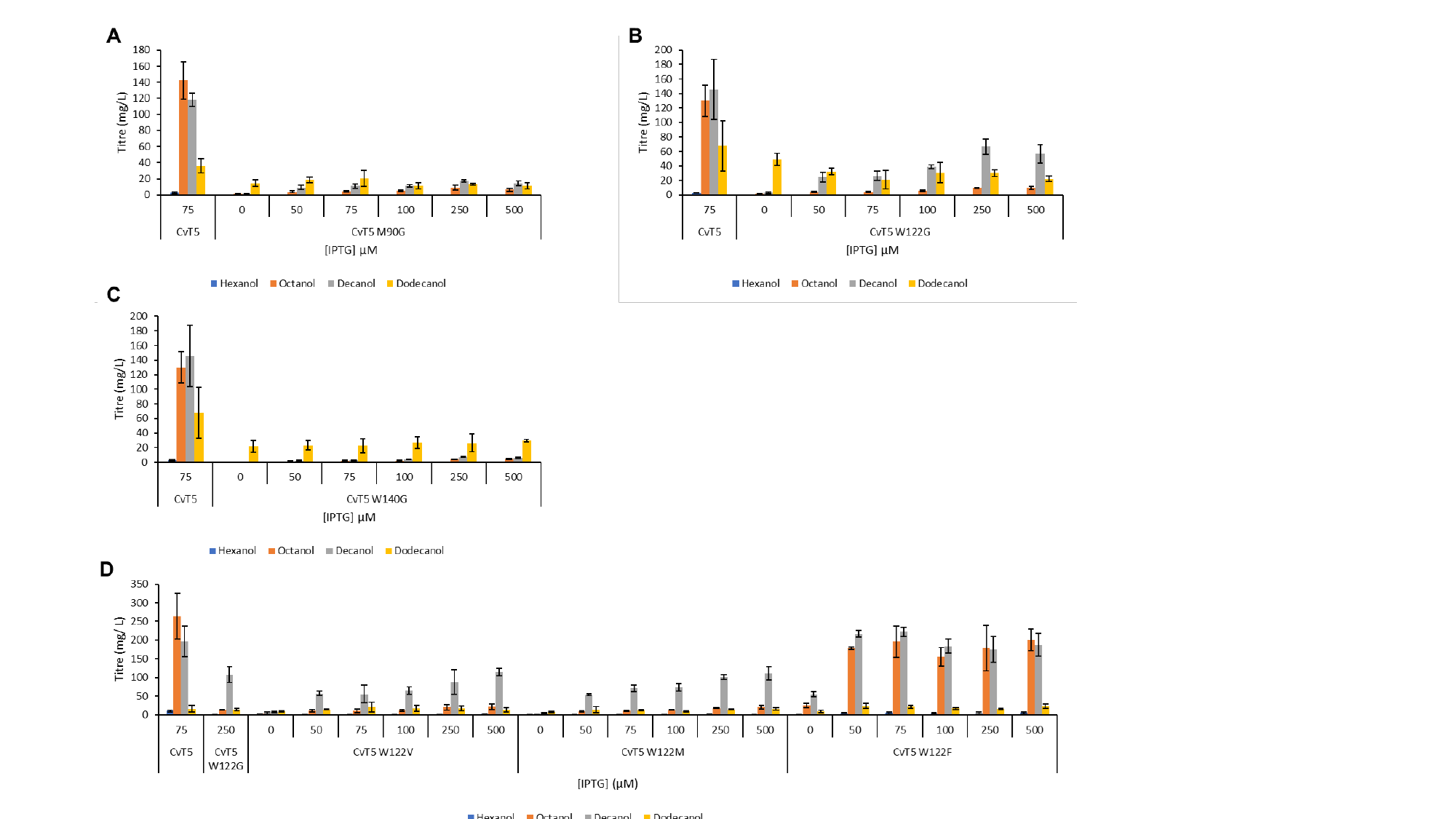

## Slide 7
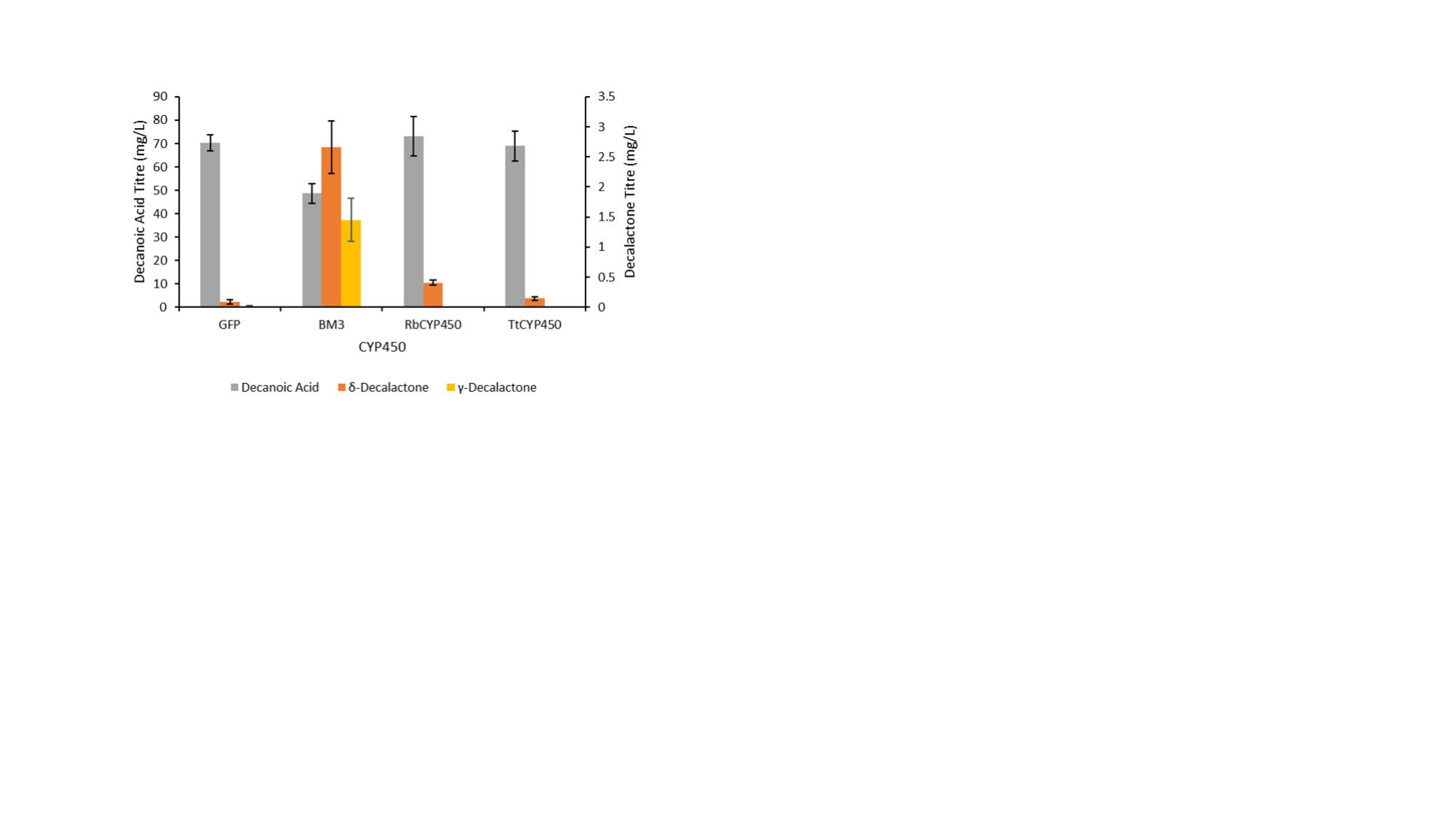

Supplement: Supplementary file 1 — Supplementary Material 1 [file 13036_2025_575_MOESM1_ESM.pptx]
